# Calculated parenteral initial treatment of bacterial infections: Bone and joint infections

**DOI:** 10.3205/id000054

**Published:** 2020-03-26

**Authors:** Mathias G. Vossen, Rainer Gattringer, Florian Thalhammer, Matthias Militz, Gunnar Hischebeth

**Affiliations:** 1 Medizinische Universität Wien, Universitätsklinik für Innere Medizin I, Klinische Abteilung für Infektionen & Tropenmedizin, Allgemeines Krankenhaus Wien, Vienna, Austria; 2 Institut für Hygiene und Mikrobiologie, Klinikum Wels Grieskirchen, Wels, Austria; 3 Klinische Abteilung für Infektiologie und Tropenmedizin, Medizinische Universität Wien, Vienna, Austria; 4 Abteilung für Septische und Rekonstruktive Chirurgie, BG-Unfallklinik Murnau, Germany; 5 Institut für Medizinische Mikrobiologie, Immunologie und Parasitologie, Universitätsklinikum Bonn, Germany

## Abstract

This is the 10^th^ chapter of the guideline “Calculated initial parenteral treatment of bacterial infections in adults – update 2018” in the 2^nd^ updated version. The German guideline by the Paul-Ehrlich-Gesellschaft für Chemotherapie e.V. (PEG) has been translated to address an international audience.

This chapter deals with bacterial Infections of bones, joints and prosthetic joints. One of the most pressing points is that after an initial empirical therapy a targeted antimicrobial which penetrates well to the point of infection and is tolerated well over the usually long duration of the therapy is chosen.

## Zusammenfassung

Dies ist das zehnte Kapitel der von der Paul-Ehrlich-Gesellschaft für Chemotherapie e.V. (PEG) herausgegebenen S2k Leitlinie „Kalkulierte parenterale Initialtherapie bakterieller Erkrankungen bei Erwachsenen – Update 2018“ in der 2. aktualisierten Fassung.

Diese Kapitel befasst sich mit bakteriellen Knochen- und Gelenks- sowie Implantatinfektionen. Dabei sollte nach einer kalkulierten Initialtherapie nach Möglichkeit immer auf eine keimgerichtete Therapie mit einem gut an den Infektionsort penetrierenden und über die üblicherweise lange Therapiedauer gut verträglichen Antibiotikum umgestellt werden.

## Introduction

Early diagnosis and adequate treatment are critical for progression and prognosis of bone and joint infections. These include radical surgical debridement, sequestrectomy or joint synovectomy, stabilization of fracture/pseudarthrosis and skin-soft tissue defect repair. Antibiotic treatment is indicated (Table 1 (Tab. 1)). 

If possible, attempts should be made to obtain sample material for microbiological processing. This is especially true in the case of chronic osteomyelitis, in which there is no acute need for action and thus diagnosis should be the top priority. The removal of sample material from the bone, preferably before the start of antimicrobial therapy or after an antibiotic break lasting at least 2 weeks, is considered standard clinical procedure. If, in the case of acute osteomyelitis, calculated initial treatment has been started, the change to targeted treatment should take place as soon as the pathogens have been identified and the results of sensitivity testing are available [1], [2].

Traditionally, initial high-dose parenteral treatment is recommended. Sequential therapy is possible if adequate drug levels can be ensured with oral medication. Based on the available studies, initial high-dose oral treatment with clindamycin or trimethoprim/sulfamethoxazole can be carried out in cases of chronic osteomyelitis [3]. Doxycycline and tigecycline have very different penetration depending on the type of infected bone and should therefore only be used in special cases. A combination of rifampicin with fusidic acid has been used for a long time without any issues but can lead to low levels of fusidic acid through induction of CYP3A4 and should be used with restraint until further notice due to the risk resistance induction [4]. In the first two weeks, additional administration of fosfomycin may be considered, regardless of the origin of osteomyelitis [5], [6], [7]. The possibilities, advantages and disadvantages of out-patient parenteral antimicrobial therapy (OPAT) should be discussed with the patient [8].

With regard to the choice of therapeutic agent, there appears to be no difference regarding treatment success or recurrence rate between bacteriostatic and bactericidal antimicrobial agents due to the long duration of treatment which is necessary in any case [9].

## Hematogenous osteomyelitis

In osteomyelitis, there is an infection of the medullary cavity, with a distinction made between a post-traumatic/post-operative origin and a hematogenous origin. The pathogen spectrum in the hematogenous form varies according to age. In adulthood, monoinfections are dominated by *Staphylococcus* *aureus*, streptococci or enterobacteria.

Depending on the expected pathogen and local resistance situation, calculated treatment is started with a group 2 or 3 cephalosporin in combination with clindamycin or an aminopenicillin/beta-lactamase inhibitor (BLI). For staphylococcal infections, monotherapy with flucloxacillin or a group 1 cephalosporin is preferable. In principle, for flucloxacillin, as with all beta-lactams, continuous or more frequent dosing is preferable; however, experience shows that splitting the daily dose into 3 equal single doses (“q8” dosing) also results in adequate treatment success. In enterobacteria with resistance mechanisms, such as the formation of AmpC or extended spectrum beta-lactamases (ESBL), even a group 4 cephalosporin (only effective in case of infections by AmpC-producing pathogens) or a carbapenem must even be considered. The additional use of fosfomycin can be beneficial, especially in the first two weeks of treatment. As an alternative to beta-lactams, moxifloxacin can be used as monotherapy or a group 2 or 3 fluoroquinolone in combination with clindamycin.

Due to its high penetration into the bone, fusidic acid is also a good combination partner in the treatment of *Staphylococcus* *aureus* osteomyelitis. 

The combination of fosfomycin with a cephalosporin may be considered, especially in complicated cases (for instance severe spondylodiscitis) [10].

## Spondylodiscitis

Spondylodiscitis is a particular form of hematogenous osteomyelitis. As the name implies, in this form not only the vertebral body itself is infected but also the disc. Here, too, Gram-positive cocci dominate as infectious agents, in particular *Staphylococcus* *aureus* (including MRSA). In cases where only the bone is affected, the disease is often associated with *Mycobacterium* *tuberculosis*. In normal cases, treatment duration of 6 weeks is sufficient [11], [12]. The choice of antimicrobial agents is the same as for hematogenous osteomyelitis, as the pathogen is usually unknown before treatment starts. In addition to multiple blood cultures, an attempt to isolate the pathogens should also be made using CT-assisted puncture. If the origin is unclear, a focus search should be carried out. It should be noted that – although this was only noted in a retrospective analysis – there is a significantly higher recurrence rate with vancomycin compared to daptomycin in MRSA spondylodiscitis [13]. In addition, an orthopedic or spinal surgery consultation should always be requested to assess the need for additional surgical restoration and/or fitting of a corset to prevent vertebral body compression.

## Post-traumatic/post-operative osteomyelitis

This develops post-traumatically (through direct contamination during trauma) or post-operatively (intra-operatively). *Staphylococcus* *aureus* is also the underlying pathogen in many cases, especially in post-operative osteomyelitis. However, mixed infections with streptococci, Enterobacteriaceae and anaerobes are also common. In some cases, *Pseudomonas* *aeruginosa* may also be considered the underlying cause in post-traumatic osteomyelitis.

Treatment must commence as early as possible in surgical debridement, the removal of any foreign bodies with bone stabilization and calculated initial antibiotic treatment. In any case the material obtained during debridement should be sent for microbiological analysis. For antimicrobial treatment, aminopenicillin/BLI (i.v.), a cephalosporin of group 2 or 3 (i.v.) or clindamycin are recommended. Where there is high risk of multidrug-resistant staphylococci, daptomycin, linezolid, teicoplanin or a high-dose cephalosporin of group 5 can be used. For the latter, however, there is little experience so far; there is in particular a question of possible resistance development due to the necessarily long duration of treatment [14], [15]. The use of vancomycin cannot be recommended because of its low bone penetration and simultaneously limited maximum serum levels due to the nephrotoxic risk. Rifampicin shows good penetration, both in the bones and in biofilms. It is therefore generally suitable as an additional treatment option. However, its use should be limited as far as possible to the treatment of foreign body-associated infections. When using linezolid, initially a higher dosage of up to 3x 0.6 g or a front-loading strategy of 2x 1.2 g can be considered. However, the expected higher efficacy should be weighed carefully against its potentially toxic effects [16], [17], [18].

Combination with fosfomycin during the first two weeks of treatment can bring added benefits. If Gram-positive pathogens are strongly suspected, out-patient intravenous treatment with a long acting glycopeptide such as dalbavancin or teicoplanin may also be considered. If there is a suspected or confirmed infection with *P. aeruginosa*, a *Pseudomonas*-effective cephalosporin (for example ceftazidime or cefepime) or piperacillin/tazobactam should be used.

In chronic osteomyelitis, the infected bone and any existing implants must be removed. Targeted antibiotic treatment should be carried out [19], [20].

## Sternal osteomyelitis

Sternal osteomyelitis generally occurs post-operatively as a complication of a sternotomy but occasionally it can have a hematogenous origin. In essence, it is caused by *Staphylococcus* *aureus* or coagulase-negative staphylococci, which are often multiresistant. However, there are also individual reports of fungal-associated sternal infections [21], [22]. Antibiotic treatment is initially high-dose with an isoxazolylpenicillin or a cephalosporin of group 1 or 2 in combination with clindamycin or fosfomycin. For infections with MRSA or methicillin-resistant coagulase-negative staphylococci, such as *Staphylococcus* *epidermidis*, the use of daptomycin or linezolid is recommended [23]. Again, the use of a high-dose cephalosporin of group 5 is worth considering but so far this has hardly been tested [14].

## Bacterial arthritis

The main cause of bacterial arthritis is iatrogenic infection. With regard to the prognosis, early infection should be differentiated from late infection. The pathogens are usually staphylococci or beta-hemolytic streptococci of groups A, B, C and G. Other pathogens such as Enterobacteriaceae and gonococci are rare. In addition to immediate surgical treatment, also with confirmation of diagnosis, calculated initial antibiotic treatment similar to that of post-operative osteomyelitis is recommended. If the pathogen is confirmed through a preceding puncture, treatment with antibiotics only may be considered sufficient for rare infections caused by salmonella or gonococci.

## Endoprosthesis/foreign body-associated infections

60–70% of endoprosthesis infections develop within the first two years following implantation [24]. *Staphylococcus* *aureus* and coagulase-negative staphylococci are the most common causes. Polymicrobial infections occur in about 15% of cases. Basically, there is a high risk of biofilm formation not only in endoprostheses but in any foreign body, especially of coagulase-negative staphylococci a high proportion of whom show resistance to multiple classes of antibiotics [25]. *Propionibacterium* *acnes* is found mainly in infected shoulder prostheses but it is ultimately difficult to rule out culture contamination [24].

Expansion or changes after radical surgical debridement and, ideally targeting specific pathogens, treatment with maximally high doses of antibiotics are the treatment of choice [26]. Extracted foreign bodies can be treated with ultrasound to improve the sensitivity of pathogen detection [27]. This can lead to pathogen diagnosis even in prosthesis loosening primarily interpreted as aseptic [28]. In early prosthesis infections (within the first 2[–4] weeks), even replacement of removable surfaces and surgical debridement followed by three to six months of biofilm-active drug treatment (rifampicin or high-dose daptomycin) may result in healing (“debridement, antibiotics, and implant retention [DAIR]”) [29], [30], [31], [32], [33]. Streptococcal infections show a greater tendency for healing than infections with *Staphylococcus* *aureus* [34]. Even in single-stage implant replacement, biofilm-active treatment should be administered over three months [35]. Following initial four to six-week intravenous treatment with an aminopenicillin/BLI, a group 1 or 2 cephalosporin or a glycopeptide, each in combination with rifampicin, further oral treatment should carried out with rifampicin in combination with a suitable fluoroquinolone (preferably levofloxacin or moxifloxacin) to prevent development of resistance to rifampicin. However, there is only one study about a small randomized controlled trial on the use of ciprofloxacin plus rifampicin for early prosthesis infections [36]. Nonetheless, group 3 fluoroquinolones (levofloxacin) and group 4 (moxifloxacin) should probably be considered superior to ciprofloxacin (group 2) [36], [37]. The correct dosage of rifampicin is uncontroversial. While often a dosage of 2x 0.3–0.45 g per day is recommended, there are also pharmacokinetic arguments for a dosage of 1x 0.6 g [36], [38], [39]. The combination of daptomycin plus rifampicin showed very good efficiency in tests with rats [40]. Fosfomycin has shown very good efficacy in the treatment of implant-associated MRSA osteomyelitis in animal tests but should not be used as monotherapy due to the rapid development of resistance [7], [41], [42]. Linezolid was discussed as an alternative treatment for late prosthesis infections [43]. Healing rates under exclusively conservative treatment are very low.

## Notes

This is the tenth chapter of the guideline “Calculated initial parenteral treatment of bacterial infections in adults – update 2018” in the 2^nd^ updated version. The German guideline by the Paul-Ehrlich-Gesellschaft für Chemotherapie e.V. (PEG) has been translated to address an international audience.

Following the publication of the 1^st^ version of the guideline in German, these dosage suggestions were updated by the working group (Table 1: Recommendations for the calculated antibiotic therapy of bone and joint infections): dalbavancin 1.5 g on day 1 and day 8, sufficient for 8 weeks INSTEAD OF dalbavancin 1 g as first dose, then 0.5 g once per week as a maintenance dose or 1.5 g every 15 days.

## Competing interests

The authors declare that they have no competing interests.

## Figures and Tables

**Table 1:**
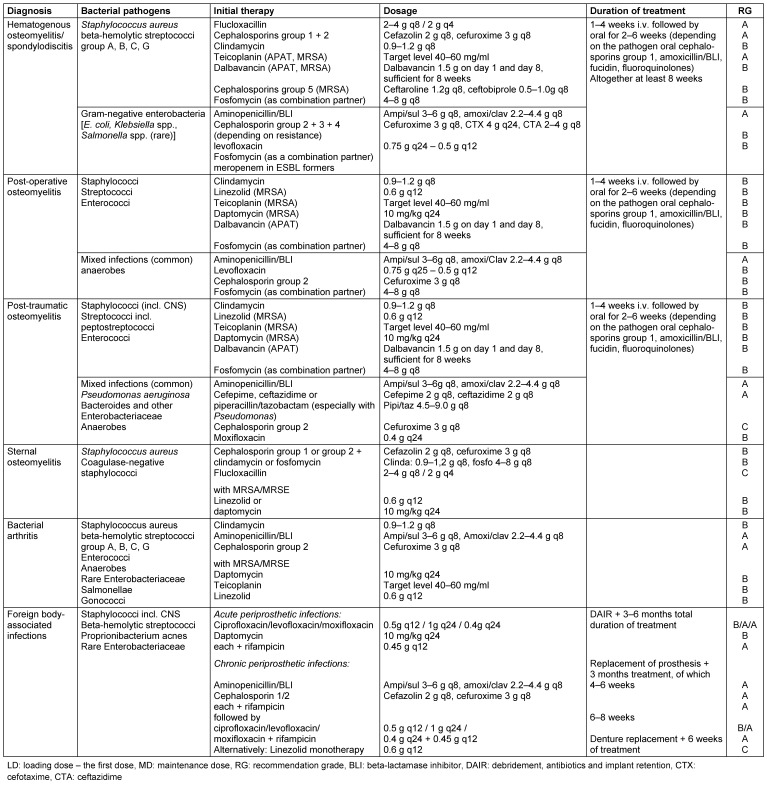
Recommendations for the calculated antibiotic treatment of bone and joint infections
